# Use of artificial intelligence in the out-of-hospital care settings: a scoping review

**DOI:** 10.1136/bmjdh-2026-000035

**Published:** 2026-05-06

**Authors:** Jamie Miles, Mike Brady, Leanne Smith, Charlotte Cotterill, Charlotte Levey

**Affiliations:** 1 The University of Sheffield Faculty of Medicine Dentistry and Health, Sheffield, South Yorkshire, UK; 2 Welsh Ambulance Services NHS Trust, Cwmbran, Wales, UK

**Keywords:** Artificial intelligence, Hospitals, Emergency Service, Hospital

## Abstract

**Background:**

Out-of-hospital services face significant challenges, including growing patient demand, workforce limitations and evolving care pathways. Artificial intelligence (AI) technologies offer potential solutions, but their application in out-of-hospital settings remains inconsistently implemented and poorly understood.

**Objective:**

To identify the types of AI technologies being applied in out-of-hospital settings, explore their purposes and implementation contexts and examine associated outcomes.

**Methods:**

Six electronic databases were searched for English-language studies published between 2013 and 2024. Eligible studies involved AI technologies in the out-of-hospital emergency services setting. Data were synthesised according to six implementation domains: system level, dispatch zone, response zone, on-scene zone, onward prognosis and inferential (insights).

**Results:**

From 236 publications, we identified diverse AI applications across the care pathway. System-level implementations (46 studies) featured AI for demand forecasting, optimal resource allocation and strategic facility location, with demonstrated improvements in coverage efficiency of 10–20%. In the dispatch zone (32 studies), AI-enhanced emergency call triage and ambulance allocation reduced response times by up to 10–20%. Response-level applications (43 studies) included intelligent traffic management and real-time route optimisation, reducing travel times by 15–30%. On-scene zone implementations (75 studies) supported clinical decision-making with cardiac arrest rhythm detection, achieving an area under the curve (AUC) values exceeding 0.90 and acute coronary syndrome prediction sensitivities of 85–90%. Onward prognosis models (19 studies) predicted patient outcomes with some AUC values of 0.80–0.90 for survival forecasting, enabling better resource allocation and early intervention. Further inferential analysis applications (21 studies) were also identified that provide higher-level insights through secondary analyses of out-of-hospital data.

**Conclusions:**

AI demonstrates significant potential across the care pathway, from operational optimisation to clinical decision support. Future development should focus on real-time adaptive systems, ethical implementation, improved data integration across the care continuum and rigorous evaluation of real-time patient outcomes. Cross-disciplinary collaboration and standardised reporting of AI implementations will be essential to realise the full potential of these technologies in improving out-of-hospital care delivery.

**WHAT IS ALREADY KNOWN ON THIS TOPIC**

- Artificial intelligence (AI) technologies show theoretical promise for enhancing out-of-hospital care services, but limited information exists about their implementation and real-world impact.

**WHAT THIS STUDY ADDS**

- This first comprehensive scoping review identifies a critical implementation gap: of 236 publications describing AI applications in out-of-hospital care, fewer than 15% report functional clinical deployments and fewer than 5% document sustained implementation with evaluation.

**HOW THIS STUDY MIGHT AFFECT RESEARCH, PRACTICE OR POLICY**

- Our findings suggest an urgent need to shift focus from developing novel AI applications to implementing and evaluating existing ones, addressing key barriers, including technical integration challenges, regulatory hurdles, evidence requirements and organisational change management.

## Introduction

### Rationale

Out-of-hospital care spans ambulance services, NHS 111 and out-of-hours provision, delivering over 67 million contacts annually in the UK amid intensifying pressures such as ageing populations, workforce constraints, limited community alternatives, growing elective backlogs and underuse of emerging technologies.^1–3^ The move away from ‘transport-to-hospital by default’ has exposed system-wide interdependencies requiring coordinated responses.^4^ Artificial intelligence (AI) offers means to optimise performance for better outcomes. Existing reviews have been conducted; however, their findings are either too focused (such as on trauma only), or too broad with scant implementation analysis.^5 6^ One review focused on low- and middle-income country contexts without high-income perspectives.^7^ Despite rapid growth, a whole-system synthesis is missing.

### Objectives

Current ambitions for AI include enhanced triage processes, predictive analytics, telemedicine applications and remote patient monitoring.^8^ However, significant knowledge gaps persist regarding the impact of AI in healthcare, necessitating an improved understanding of where these tools are being used, how they are implemented, and their effects on stakeholders.^9 10^

This scoping review aims to address these knowledge gaps by (1) identifying the types of AI technologies being applied in out-of-hospital settings, (2) exploring the purposes for which these technologies are used and (3) examining the outcomes associated with their implementation.

## Methods

### Protocol and registration

The research team developed and approved a protocol but did not formally register it.

### Eligibility criteria

Studies were included if they: (1) related to out-of-hospital emergency care settings (eg, ambulance services); (2) used, piloted or explored AI technologies; (3) were published in English between 2013 and 2024; and (4) appeared in peer-reviewed journals, conference proceedings or reputable preprints. Studies not meeting these criteria were excluded.

#### Information sources and search

Six electronic databases were searched: CINAHL, MEDLINE (via PubMed), IEEE Xplore, ACM Digital Library, Scopus and arXiv. The search strategy combined terms related to out-of-hospital care and AI/machine learning (ML). The full search strategy can be found in the online supplemental material.

10.1136/bmjdh-2026-000035.supp1 Supplementary data

#### Selection of sources of evidence

Records were imported into Rayyan software for screening and de-duplication. Titles and abstracts were independently screened by at least two reviewers, followed by full-text screening for eligible studies.^11^ Disagreements were resolved through discussion and consensus. Where review articles were identified, these were treated as a single included record. We did not extract or double count the individual studies within such reviews in our total of 236 included studies. We compared the reference list of all review articles to the extracted individual articles. We removed any individual articles that were found in these lists to prevent duplication.

#### Data items

Data extracted included study details (author, year, country), AI technology used, the setting type, primary aim and methodology, outcomes assessed and key findings/conclusions. Two reviewers performed data extraction independently, with discrepancies resolved through team discussion. The data is available online.^12^

#### Critical appraisal of individual sources of evidence

A formal critical appraisal was not conducted, consistent with scoping review methodology. However, study quality and relevance were assessed based on study design, completeness of reporting and potential biases. Studies with incomplete data or unclear methodologies were flagged for cautious interpretation.

#### Synthesis of results

Data were synthesised using a narrative approach, mapping AI applications to their intended functions within out-of-hospital care and were categorised into five domains: system level, dispatch zone, response zone, on-scene zone and onward prognosis. Quantitative and qualitative findings were summarised according to these domains, effectiveness and implementation barriers.

#### Patient and public involvement

Patients or the public were not involved in the design, or conduct, or reporting, or dissemination plans of our research.

## Results

### Selection of sources of evidence

**Figure 1:**
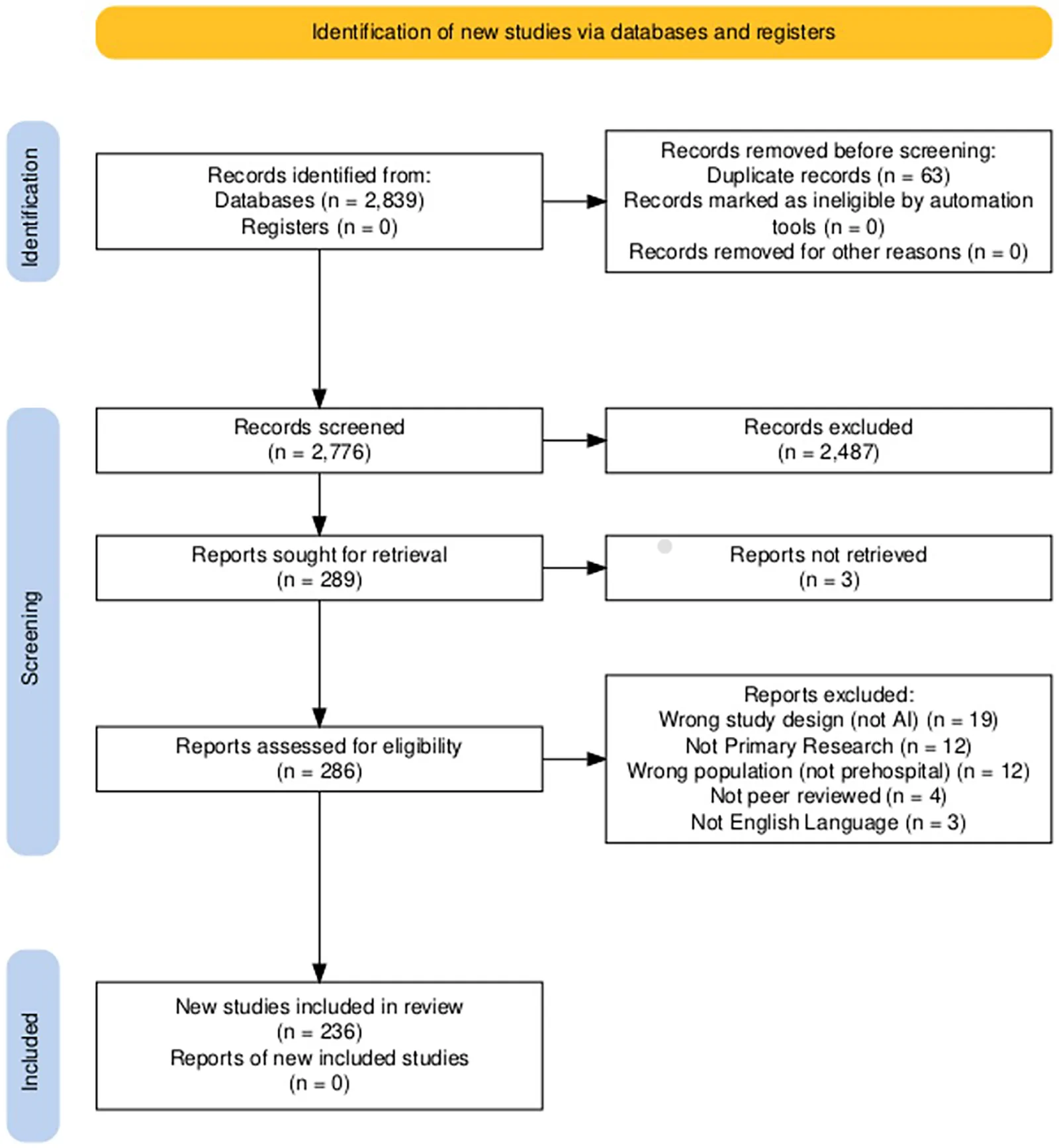
PRISMA-ScR flow diagram. AI, artificial intelligence; PRISMA-ScR, Preferred Reporting Items for Systematic Review and Meta-Analysis extension for Scoping Reviews.

The search yielded a total of 2776 records. After removing duplicates, 289 unique studies were screened at the title and abstract levels. Following a full-text review, 236 studies met the inclusion criteria and were included in the final synthesis. The study selection followed the PRISMA-ScR (Preferred Reporting Items for Systematic Review and Meta-Analysis extension for Scoping Reviews) flow diagram methodology (figure 1).^13^

### Characteristics of sources of evidence

**Figure 2:**
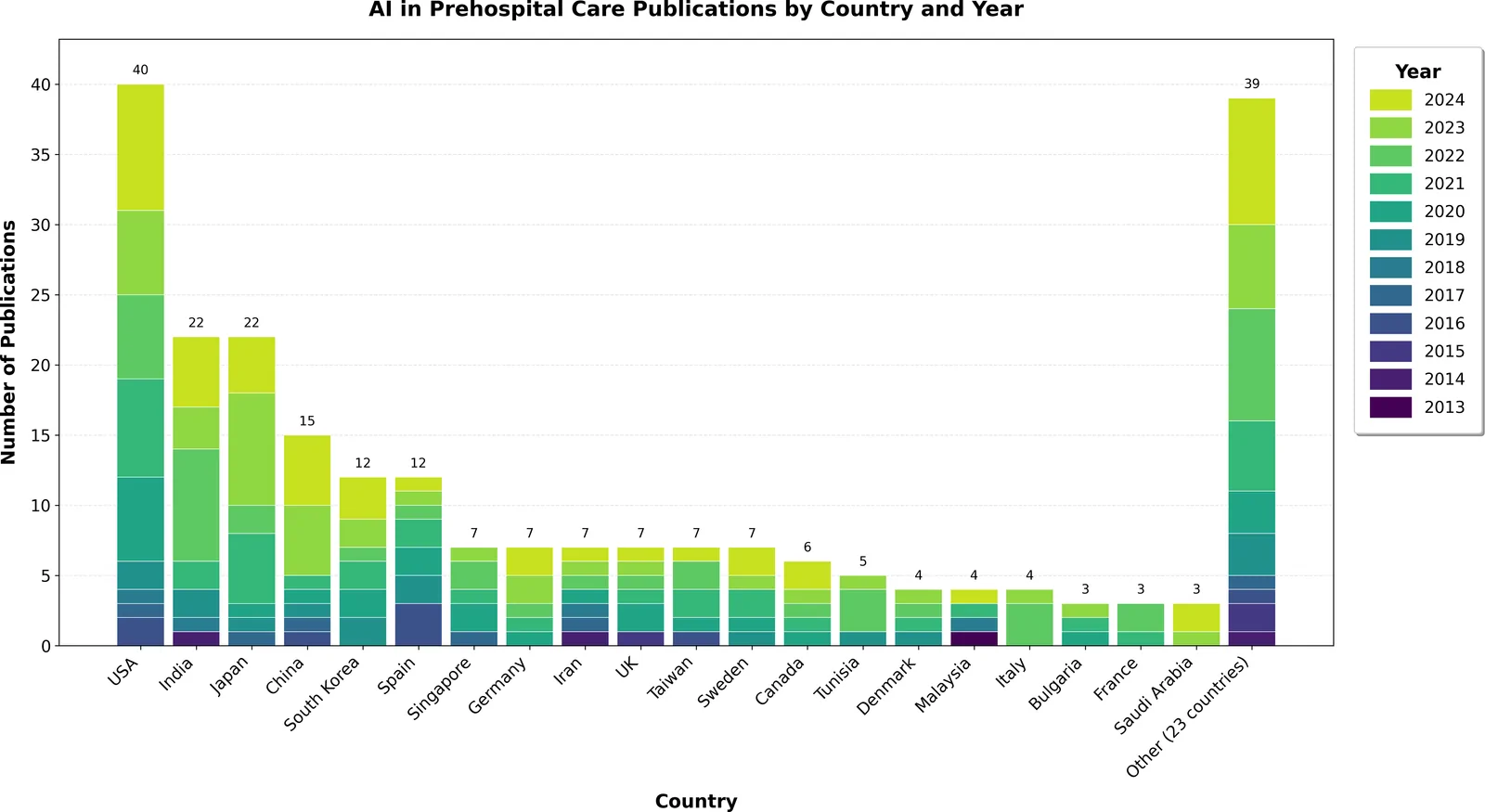
Publications by country and year (top 20+ others, 2013–2024). AI, artificial intelligence.

The 236 publications originated from 41 countries, with Asia (41%), Europe (28%) and North America (19%) being the dominant regions, led by the USA, Japan and India (figure 2).

### Synthesis of results

In out-of-hospital care, a generally linear pathway was identified that begins when someone accesses help and ends when they receive definitive care. Our analysis revealed five distinct domains where AI is being applied:

1. System level (46 studies): Applications optimising the entire infrastructure, including demand forecasting, resource planning and strategic facility location.
2. Dispatch zone (32 studies): Tools for initial triage, resource decision-making and allocation during the call-handling phase.
3. Response zone (43 studies): Technologies supporting vehicle routing, traffic management and alternative response modes during transit to patients.
4. On-scene zone (75 studies): Applications supporting clinical assessment, intervention and patient decision-making.
5. Onward prognosis (19 studies): Models predicting patient outcomes to inform care decisions and resource allocation.

Additionally, we identified inferential analysis applications (21 studies) that provide higher-level insights through secondary analyses of out-of-hospital data, examining trends, associations and underlying mechanisms across the pathway.

Table 1 shows a matrix of counts between the applied area and the methodological group. AI methods identified were grouped into five categories: statistical/ML, deep learning, natural language processing (NLP), evolutionary algorithms and hybrid approaches. Evolutionary algorithms refer to optimisation techniques inspired by natural selection.

**Table 1:** A matrix that shows the count of studies clustered by applied area versus methodological type

Count of level	Method group					
**Applied area**	**(1) Classical machine learning**	**(2) Deep learning**	**(3) Evolutionary**	**(4) Natural language**	**(5) Ensemble/proprietary**	**Grand total**
System level	14	17	11	0	4	46
Dispatch zone	5	3	13	2	9	32
Response zone	10	21	8	0	4	43
On-scene zone	35	21	3	3	13	75
Onward prognosis	9	4	0	0	6	19
Inferential analysis	8	6	0	6	1	21
**Grand total**	**81**	**72**	**35**	**11**	**37**	**236**

The findings are presented across five domains: system-level applications, dispatch, response, on-scene care and onward prognosis. Each domain is described using a common structure to facilitate comparison, beginning with the scope of applications identified, followed by illustrative examples of current use and future directions. Some sections also have an accuracy and ethical considerations where applicable.

Performance estimates should be read in context. Across studies, dataset size and class balance varied widely and were frequently under-reported; external validation and human comparators were inconsistently provided. To aid interpretation without overburdening the narrative, we only include concise point estimates reported in the studies.

## The system level

### Scope and focus

46 studies focused on larger-scale, macro-level applications of AI in emergency medical services (EMS).^14–26^ ‘System-level AI’ refers to methods designed to optimise the whole system infrastructure across regions or jurisdictions, focusing on aggregate-scale decisions rather than individual incidents. Key components include forecasting,^15 22 27–37^ location allocation,^16 20 23 26 38–44^ scheduling^45^ and resource management^16 17 21 22 38 44 46 47^ - requiring ML, optimisation, simulation or hybrid algorithms to handle complexity and uncertainty.

#### Current applications

##### Demand forecasting

For out-of-hospital care, proactively modelling spatiotemporal demand is fundamental to effective prepositioning and staffing. System-level studies use time-series and ML forecasting to predict ambulance/EMS call volumes across time and geography, enabling more responsive deployment.^15 22 27 30 32–34^ Others focus on specific purposes: Martin *et al* and Ramgopal *et al* employ techniques to anticipate demand surges, Lin *et al* explore deep learning for short-term call volume forecasting and Kang *et al* predict critical care needs.^30 32 34 48^

##### Ems resource and station location

After understanding demand patterns, the strategic focus shifts to balancing geographically positioned resources. Studies tackle location-allocation problems, aiming to optimise static resources (stations, defibrillator sites) and dynamic resources (ambulances, drones) to maximise coverage or minimise response times.

Approaches include:

- Integer linear programmes and mixed-integer models.^20 41 43^
- Genetic algorithms, particle swarm optimisation and artificial immune systems.^16 17 38 40 42 49^
- Multi-objective approaches balancing cost, coverage and response times.^44^

##### Deployment and routing beyond individual dispatch

Several studies cover strategic routing for uncertain demand and geographical constraints, including routing/scheduling constraints and congestion-aware redeployment,^16 38 45^ and dynamic/robust location-allocation under uncertainty,^20 39^ with emerging work considering drone-based response infrastructure.^43^

##### Integration of climate and environmental factors

Some research incorporates external phenomena such as heatwaves and meteorological conditions into forecasting models,^28 34 36 37^ reflecting the need to model environmental triggers alongside temporal patterns and other contextual determinants.^33 50^

##### Future directions

##### Real-time adaptive systems

Several system-level studies described dynamic (re)planning approaches that combine forecasting with repeated re-optimisation of coverage and asset relocation/repositioning, including algorithm portfolios with adaptation/learning mechanisms and congestion-aware relocation evaluated via simulation.^15–17 20 22 38^

Geographical equity and rural access were explicitly foregrounded in at least one location-allocation formulation addressing sparse station coverage and long travel times.^40^

## Climate resilience and disaster preparedness

System-level forecasting studies incorporated meteorological and heatwave features to predict ambulance call/transport volumes, including regional/national modelling and deep learning using weather covariates.^28 37 50^

Separately, dispatch data analyses during COVID-19 were used to characterise demand shifts and temporal patterns relevant to surge contexts.^35^

## Collaboration and standardisation

A small subset of optimisation studies reported benchmark-based comparisons and/or algorithm portfolio evaluations, supporting structured comparison of alternative solution approaches across problem instances.^20 32 49^

## Equity and ethical AI

At system level, equity-related treatment was most clearly evidenced through rural–urban access considerations in location-allocation modelling.^40^

A separate system-level predictive study integrated social determinants alongside clinical factors (rather than focusing on allocation objectives).^14^

### *The* dispatch zone

### Scope and focus

There were 32 studies applicable to the dispatch zone, examining how AI supports decision-making and resource allocation during emergency medical dispatch. Dispatch-level AI addressed two interlinked processes: (1) call handling, including call classification and triage (eg, ML-supported recognition of cardiac arrest and dispatch triage modelling), and (2) resource deployment, including assignment/routing and policy designs that balance coverage, response times and crew workload.^51–55^

Across studies, dispatch models combined historical dispatch/call data with predicted clinical urgency and used spatial/travel-time constraints to inform allocation and routing decisions, including congestion-aware or policy-aware dispatch/relocation formulations and vehicle-routing optimisation.^51 55–59^

## Current applications

### Triage and call classification

Several studies explore ML or NLP for predicting call severity and prioritisation.^52 53 55 60–64^ Chin *et al* discuss early recognition of emotional cues in out-of-hospital cardiac arrest (OHCA) calls, enabling prioritisation of emotionally unstable callers.^65^ Campo-Ávila *et al* proposed data mining techniques that aided telephone operators in detecting suicidal behaviour.^66^

## Ambulance location and deployment optimisation

A major focus is optimal ambulance placement and relocation, anticipating demand surges.^51 58 59 67–69^ Babaei and Shahanaghi adopt predictive models for demand forecasting and dynamic redeployment to minimise response times.^51^

Mathematical models combined with heuristic algorithms support decision-making. Ravandi *et al* propose a multi-objective formulation considering ambulance allocation and technician capability.^58^ McCormack and Coates present simulation models demonstrating increased OHCA survival rates without additional resources.^69^ Hussein, Frikha and Rahebi employ optimisation algorithms showing superiority over other methods in busy urban centres.^57 69^

## Predictive routing and real-time navigation

Routing-focused studies modelled travel-time constraints and patient priority, including bi-objective routing formulations and smart-city routing optimisation approaches.^51 57 70^

## Accuracy and success of dispatch-level AI

### Performance metrics

Evaluating success varies between development and real-life implementation. Research models evaluate success using accuracy, sensitivity, specificity or area under the curve.^52 55 60–62 64–66^ Chin *et al* report 80–90% accuracy in distinguishing cardiac arrest calls from less urgent events.^65^

In real-life settings, systems evaluate success via response times and coverage ratios.^58 59 67–69^ Dynamic redeployment studies often achieve 10–20% reductions in mean response time relative to static placement. Multi-objective models demonstrate better coverage with fewer ambulances.^58 68^

## Future directions

### integration with clinical systems

A subset of dispatch-zone studies extended beyond call classification to downstream clinical decision points, including COVID-19 case identification at call triage to support subsequent service pathways and hospital resource planning, prediction of patient conveyance using call data and injury severity prediction to support prehospital decision-making.^62 63 71^ Together, these studies illustrate dispatch models being developed in ways that can be linked to on-scene assessment and onward care processes.^55 62 63 71^

### Equity and bias considerations

Dispatch studies applying speech-derived features (eg, phonetic characteristics and automatic speech recognition) for time-critical condition recognition demonstrate that audio and language signals are being used as predictive inputs in dispatch decision support.^61 72^ In addition, error-focused analysis has been used to characterise false positive/false negative outputs from cardiac-arrest call-recognition models, providing an empirical basis for examining performance variability in dispatch deployments.^61 73^

### System integration and evaluation

Several studies operationalised dispatch as part of a coupled optimisation problem, modelling dispatch and routing under uncertainty and integrated location-dispatch-relocation formulations with revised dispatch policies and multi-objective trade-offs.^51 58 59^ Prospective evaluation designs were uncommon; however, one study tested ML support for dispatcher recognition of OHCA in a randomised clinical trial setting.^52^

## The response zone

### Scope and focus

There were 43 studies in the response zone focusing on real-time or near-real-time interventions and decision-making within the emergency response process. These approaches typically aim to streamline en-route care, pre-arrival scheduling and dynamic control systems—serving as a bridge between initial dispatch decisions and on-scene interventions.^74–78^

## Current applications

### Smart traffic management systems

There have been multiple studies using AI to create ‘green corridors’ for ambulances. These typically integrate with traffic light signals to detect approaching sirens. The most prominent method is the convolutional neural network. Using this method, Mathiane *et al* accounted for traffic density and route prioritisation to derive an algorithm, which resulted in a 96% accuracy in classifying emergency vehicles.^79^ Fuzzy-logic traffic-control models for emergency response detected priority vehicles (including under occlusion) with high reported accuracy and used fuzzy inference to adapt signal timing—extending green phases by up to ~50% and prioritising emergency vehicles under varied traffic conditions.^80 81^ Traffic-signal prioritisation prototypes reported measurable reductions in emergency-vehicle intersection delay in simulated or testbed environments.^82–84^

## Route optimisation

A second cluster addressed routing and scheduling using advanced optimisation: memetic algorithms for patient transport scheduling, Tabu Search-based hyper-heuristics for ambulance routing variants, and swarm/bio-inspired pathfinding methods for routing in complex settings.^85–88^

Several studies additionally modelled travel-time uncertainty and correction using ML approaches to improve route-time estimation and navigation decisions.^89 90^

### Alternative response mechanisms

Drone-enabled response for OHCA was modelled using data-driven simulation and network design approaches, including genetic-algorithm positioning strategies and coverage modelling.^77 91^ A further study used ML to inform dispatch of drone-delivered defibrillators.^92^

Connectivity-focused work examined communication reliability for connected ambulance response, including 5G-enabled handover and connected routing/protocol proposals for emergency health contexts.^74 93^

## Future directions

### Validation and generalisability

Across traffic-management and drone-response studies, authors repeatedly noted the need for larger-scale field evaluation and testing across diverse geographic/traffic contexts to establish external validity beyond simulation and prototype conditions.^77 79 82 91 92 94^

Two traffic-management studies explicitly foregrounded broader data-collection and modelling capability to support heterogeneous urban traffic environments.^95 96^

One study additionally proposed continuous adaptive control with real-time learning as a route to improved performance under dynamic conditions.^76^

### Equity and implementation considerations

Equity-oriented work was represented by a fairness-focused routing formulation within a disaster-response context.^97^

Implementation constraints described across connected-response and drone/traffic interventions included feasibility considerations linked to infrastructure requirements and operational integration (eg, connected-road systems and communications reliability).^74 78 91 93^

## The on-scene zone

### Scope and focus

Across the on-scene literature, AI was applied to predict and classify diagnoses, identify clinical states and to provide real-time decision support using physiological signals (notably ECG), vital signs and structured clinical observations captured in out-of-hospital care.^98–103^

There were 75 studies in the ‘on-scene zone’ category involving immediate decision support for interpreting patient vitals, supporting resuscitation choices and supporting urgent transport and disposition decisions; this total includes review articles, each counted as a single record.

Several studies explicitly targeted disposition and destination decisions, including models for admission/conveyance prediction and tools supporting stroke pathways and navigation.^104–108^

## Current applications

### Cardiac arrest recognition and intervention

A prominent cluster focused on OHCA and resuscitation decision support, including prediction of return of spontaneous circulation, shockable rhythm identification, pulse detection from ECG and related treatment-relevant classification tasks.^102 109–114^ In addition, studies developed and validated models intended to inform termination or continuation of resuscitation and related prognostic judgements in the prehospital setting.^113 115^ Multiple works addressed monitor–defibrillator decision support, including shock advisory or rhythm-analysis algorithms evaluated against clinician performance or existing approaches.^116–118^

### ECG interpretation and acute coronary syndromes

Several studies evaluated AI-enabled processing of prehospital ECGs for acute coronary syndrome (ACS)/myocardial infarction (MI) identification and related risk stratification, including algorithms reporting improved specificity or strong diagnostic discrimination relative to comparators.^99–101 119^ A related strand used ECG monitoring features to support prediction of downstream clinical *outcomes or disposition*.^120^

### Triage and immediate diagnostic support

A further set of studies developed models for prehospital triage and early risk stratification, including field-triage enhancement and general emergency triage models using demographics, chief concern, vital signs and clinical history.^98 106 121 122^ Condition-specific on-scene decision support included stroke-focused tools (symptom grading and destination-relevant prediction) and a navigation/pathway tool integrating clinical timelines with geospatial information.^103 104 107^

Additional work explored disposition-adjacent outcomes such as urgent dialysis need at ambulance transport decision points and prediction of ambulance conveyance.^105 108^

### Accuracy and success of on-scene AI

Studies most commonly reported performance using AUC, sensitivity/specificity and accuracy for classification tasks (eg, shockable rhythm detection and ECG-based ACS/MI identification).^99 100 102 111^ Admission or disposition-related prediction models typically reported discrimination/accuracy in the 80–90% range (where specified) or presented internally validated risk scores for conveyance/admission outcomes.^105 106 108 122^

A smaller subset foregrounded model calibration, internal validation and safety-oriented scoring frameworks suitable for prehospital risk stratification, including transparent or interpretable modelling choices.^107 113 123 124^ Only a limited number of studies explicitly surfaced subgroup-relevant variables (eg, deprivation and geography) within the on-scene modelling or evaluation framing.^98 123^

## Ethical considerations

Ethics-adjacent issues in the on-scene set were most often operationalised through privacy-preserving computation and through interpretability/transparent scoring rather than stand-alone ethical analyses.^101 113 123 125^

Where monitor–defibrillator decision support was evaluated, studies also reflected implicit accountability concerns by comparing algorithm outputs against human decision-making or existing advisory logic.^116–118^

## Future directions

### Research quality and design

Across the on-scene set, papers frequently emphasised the need for broader validation beyond retrospective development, including prospective evaluation and/or trial-like testing in clinically realistic settings.^99–101 115 116 118 126^

### Explainability and trust

Some studies explicitly positioned interpretability, transparent scoring or explainable output structures as important for clinical use, including interpretable model construction and safety-index style outputs.^101 113 123^

### Equity and subgroup performance

A small number of studies incorporated or evaluated factors aligned with equity-relevant stratification (eg, deprivation/geography), indicating an emerging but limited evidence base within the on-scene zone.^98 123^

## Technical implementation

Implementation-oriented work described requirements consistent with device-adjacent inference (eg, monitor–defibrillator workflows), remote/connected sensing and computational constraints, including end-to-end optimisation on constrained devices and Internet of Things (IoT)-enabled integrations.^116–118 127 128^

Several authors also described multimodal or uncertainty-aware modelling directions (eg, multimodal classification and explicit uncertainty framing).^129 130^

## The onward prognosis

### Scope and focus

19 studies examined onward prognosis, forecasting future patient states based on out-of-hospital or early clinical data. ‘Onward prognosis’ denotes the application of AI methodologies to predict a patient’s likely clinical course. These studies address outcomes including survival, neurological recovery and hospital admission, reducing many predictors for OHCA outcomes and reducing bias.^131–143^ Rather than merely classifying a patient’s present state, onward prognosis provides actionable insights into condition evolution, enabling better resource allocation and informed care decisions.^135 143–146^

## Current applications

### OHCA survival prediction

Most studies focused on OHCA, forecasting survival to discharge or related endpoints using demographic variables, resuscitation timings, initial rhythm and early clinical indicators.^132 133 136 140 141 146^ Ensemble and advanced ML approaches were common, including gradient-boosted methods and interpretable frameworks.^132 133 146^ Deep learning-based prognostic systems were also reported.^134 139^ Several studies treated neurologically intact survival/neurological outcome as a distinct endpoint alongside survival.^132 134–139^

## Short-term mortality and neurological outcomes

One study developed a prospective model predicting short-term mortality among a broad prehospital cohort using early physiological parameters.^142^ A separate study modelled prehospital stroke triage strategies by predicting outcomes under alternative transfer pathways to specialist centres.^143^ Unsupervised learning was used in one OHCA study to identify clusters/phenotypes relevant to outcome heterogeneity.^147^

## Hospital admission forecasting

A smaller subset addresses whether patients require hospital admission or can be safely discharged. Strum *et al* developed an ensemble model for forecasting hospital admission from out-of-hospital data to streamline patient flow and reduce overcrowding.^145^

## Accuracy and success of predictive models

### Reported performance metrics

Across the set, studies primarily reported discrimination using AUC, alongside sensitivity/specificity and related classification metrics.^132 136 139–141 146 148^ Where stated in the studies, OHCA-oriented models reported AUCs spanning approximately 0.87–0.96, depending on the outcome and model.^136 139–141 146^ Hospital admission prediction reported discrimination around AUC ~0.77.^145^

## Future directions

Across the onward-prognosis studies, recurring methodological priorities included external validation/generalisation, calibration and interpretability/transparent risk scoring for operational use.^134 135 141 146 148^

Several studies already extended beyond binary survival by explicitly modelling neurological outcome, indicating a move towards more functionally meaningful endpoints within this zone.^132 134–136 139^

## The inferential analysis

### Scope and focus

There were 21 inferential analysis studies using secondary analyses of prehospital datasets to quantify associations, trends and mechanisms underpinning EMS activity and outcomes, including disparities in OHCA survival, environmental drivers of demand and time-interval effects across the prehospital pathway.^149–153^

## Current applications

### Social determinants of health and disparities

One cohort analysis quantified racial and socioeconomic disparities in OHCA survival.^151^ NLP-based studies additionally used prehospital free text and documentation patterns to characterise neighbourhood and contextual factors relevant to prehospital intervention quality and variation.^154 155^

## Public health and population-level phenomena

EMS data can examine broader public health issues. Two studies used paramedic/EMS records for opioid overdose surveillance and case identification suitable for public health monitoring. 5 64 A further study modelled potential climate/air-pollution impacts on EMS utilisation using simulation.^150^

## Scene times and protocol efficacy

Inferential studies examined how operational time intervals and system pressures relate to outcomes, including modelling optimal on-scene time for OHCA in relation to neurological outcome and survival, and analysing how resuscitation process variables (including time components) interact in outcome models.^149 153^ Separate work quantified the effect of ambulance busyness/availability on response-time performance.^152^

## NLP and data extraction

Several studies developed or evaluated NLP and information-extraction methods to link and structure EMS data—linking EMS and emergency department (ED) records, applying named-entity recognition for prehospital trauma narratives, and automating entity resolution to improve EMS information integration.^156–159^ NLP/ML was also used to classify patient subcohorts from ambulance attendances to support population-level characterisation and prevention-oriented analyses.^145 160^

## Future directions

Across inferential studies, a consistent methodological direction was richer data linkage and integration (eg, EMS–ED linkage, entity resolution and scalable extraction from free text) to support stronger cross-sector analyses.^156–159^ A parallel direction was combining EMS logs with social and environmental context (eg, Social Determinants of Health documentation, neighbourhood factors and pollution/climate signals) to support more explanatory models of EMS demand and outcome variation.^150 152–155^

**Reporting quality and completeness:** Although formal critical appraisal was not undertaken, we assessed completeness of reporting to support interpretation of findings. Many studies presented algorithmic performance metrics without sufficient detail on study design, population or clinical context. Several lacked clarities on data provenance or definitions of outcomes, and very few reported prospective or external validation. Equity and ethical considerations were only rarely mentioned. Collectively, these gaps restricted our ability to compare studies directly and underscore the need for more consistent reporting standards.

## Discussion

### Summary of evidence

This scoping review identified 236 publications describing potential AI applications across five domains of out of hospital care: system level, dispatch, response, on scene and onward prognosis. While the literature reveals numerous theoretical use cases spanning the entire pathway, a striking finding is a substantial gap between proposed applications and actual implementation in real-time clinical practice.

System level applications predominantly focus on demand forecasting and resource optimisation, with theoretical coverage improvements reported in simulation studies. Similarly, dispatch level AI models project response time reductions. On-scene applications demonstrate promising accuracy in retrospective analyses. In validation datasets, onward prognosis models predict patient outcomes well.

However, our review found that only a small fraction of these promising applications have progressed beyond conceptual models or simulations to real-world implementation. Of the 236 studies reviewed, fewer than 15% reported functional deployments in active clinical settings. Even fewer documented sustained implementation with longitudinal evaluation. This implementation gap represents one of the most significant barriers to realising the potential benefits of AI.

### The implementation gap: barriers to real-world application

A critical finding from our review is the substantial gap between AI development and practical implementation. Despite the abundance of promising research, few solutions have successfully transitioned from laboratory to clinical practice. This implementation gap appears to stem from several key barriers.

Technical challenges are common. Many studies highlight difficulties in data integration across disparate systems, with incompatible formats and interoperability issues preventing seamless information flow. Regulatory and governance hurdles also create hesitancy, as the regulatory landscape for AI in healthcare remains uncertain. Healthcare systems also require robust evidence of clinical and economic benefits before adopting new technologies. Most studies in our review provided limited evidence of real-world effectiveness, focusing instead on technical performance metrics in controlled settings. Organisational barriers are also a major factor. Successful implementation requires substantial organisational change, including workflow redesign and staff training.

## Barriers and facilitators to AI and ML adoption in out-of-hospital Care

Our review identified a range of inter-related organisational, technical, ethical and regulatory factors that shape adoption. Ethical considerations emerged prominently, particularly regarding algorithmic transparency and equity. Many advanced AI models are ‘black box’ in nature, which raises important questions about accountability and trust, especially when AI influences life-critical interventions.

Equity concerns are evident across all domains. Studies reveal how AI systems may inadvertently perpetuate or exacerbate existing disparities in emergency care access and outcomes. The lack of bias audits across most studies suggests a pressing need for ethical governance structures. Implementation challenges highlighted include data quality issues, resistance to adoption among clinical staff and the need for robust validation in diverse real-world settings. Organisational inertia and a lack of interoperability between systems further hinder adoption. Regulatory uncertainty also plays a significant role. Despite these barriers, several facilitators were identified. These include the development of real-time adaptive systems and stakeholder engagement in model design.

## The future direction

Looking ahead, the field must prioritise the development of real-time, adaptive systems. This focus should include ensuring robust multimodal integration and creating interpretable models that remain trustworthy, particularly under pressure. However, technical solutions alone will prove insufficient. Significant integration barriers mean that technical fixes will stall unless they are accompanied by parallel social and organisational transformation. To guide this, the field must also pivot its research methodology, moving from retrospective accuracy studies to prospective, pragmatic evaluations.

Above all, equity must be treated as a fundamental design constraint, not as an afterthought. This has practical implications: dispatch tools must incorporate multilingual capabilities, and on-scene decision support should adopt harm-aware thresholds. This commitment must extend to our success metrics. We must shift from tracking simple averages to measuring distributive fairness. Similarly, outcomes should be redefined beyond binary survival to encompass patient-centred outcomes that include quality of life.

In parallel, systems must be built for dynamic adaptation. With climate volatility increasingly altering demand and risk, these systems must be able to ingest new environmental signals. Finally, ethical governance, defined by transparency and continuous monitoring, must be baked in from the start.

## Limitations

This review has several limitations. Our search included only English language articles, which may have led to the omission of relevant studies. Second, the rapid evolution of AI technologies means that recent innovations may not yet be represented in the published literature. Third, the heterogeneity of study designs and reporting standards made direct comparisons challenging. Furthermore, publication bias likely favours positive results. Many studies report algorithm performance in controlled scenarios, which may not translate to real-world effectiveness.

## Conclusions

This scoping review reveals a paradox: an abundance of theoretical applications with promising performance metrics, but a scarcity of successful real-world implementations. While the literature demonstrates significant potential, the field faces a critical implementation gap that must be addressed.

Future efforts should focus on bridging this gap. This requires creating implementation science frameworks and building evidence for clinical impact through pragmatic trials in real-world settings. It will also be necessary to address interoperability challenges through standardised data formats and to develop implementation toolkits that address organisational and regulatory barriers. By shifting focus from developing novel applications to implementing and evaluating existing ones, the emergency care community can begin to close the implementation gap and harness the transformative potential of these technologies. Without this shift, the field risks accumulating an ever-growing library of promising but unused AI solutions.

## Data Availability

Data are available in a public, open access repository. The extracted study data, figure source files and analysis code underlying this article are deposited on Zenodo and permanently accessible via the DOI 10.5281/zenodo.17038584. No identifiable patient-level data are included, and no additional unpublished data are available.
